# Cascading Multi-Hop Reservation and Transmission in Underwater Acoustic Sensor Networks

**DOI:** 10.3390/s141018390

**Published:** 2014-07-02

**Authors:** Jae-Won Lee, Ho-Shin Cho

**Affiliations:** School of Electronics Engineering, Kyungpook National University, Daegu 702-701, Korea; E-Mail: jwlee@ee.knu.ac.kr

**Keywords:** multi-hop reservation, multi-hop relay, handshaking-based MAC protocol, hidden-node problem, long propagation delay, underwater acoustics, sensor networks

## Abstract

The long propagation delay in an underwater acoustic channel makes designing an underwater media access control (MAC) protocol more challenging. In particular, handshaking-based MAC protocols widely used in terrestrial radio channels have been known to be inappropriate in underwater acoustic channels, because of the inordinately large latency involved in exchanging control packets. Furthermore, in the case of multi-hop relaying in a hop-by-hop handshaking manner, the end-to-end delay significantly increases. In this paper, we propose a new MAC protocol named cascading multi-hop reservation and transmission (CMRT). In CMRT, intermediate nodes between a source and a destination may start handshaking in advance for the next-hop relaying before handshaking for the previous node is completed. By this concurrent relaying, control packet exchange and data delivery cascade down to the destination. In addition, to improve channel utilization, CMRT adopts a packet-train method where multiple data packets are sent together by handshaking once. Thus, CMRT reduces the time taken for control packet exchange and accordingly increases the throughput. The performance of CMRT is evaluated and compared with that of two conventional MAC protocols (multiple-access collision avoidance for underwater (MACA-U) and MACA-U with packet trains (MACA-UPT)). The results show that CMRT outperforms other MAC protocols in terms of both throughput and end-to-end delay.

## 1. Introduction

Underwater acoustic sensor networks (UWSNs) have begun to draw the attention of researchers because of their potential use in a wide variety of applications, such as environmental monitoring, resource investigation, disaster prevention and recovery, navigation and military surveillance [1]. To implement these applications efficiently, it is important to understand the characteristics of an underwater channel and to design an efficient media access control (MAC) protocol that allows communication nodes to access the shared channel.

Unlike in terrestrial wireless communication, radio signals suffer severe path losses in the underwater environment; therefore, acoustic signals are typically employed in underwater communication. However, underwater acoustic links also suffer path losses, time-varying multi-path fading, motion-induced Doppler spread and aquatic noise [2]. Accordingly, when designing an underwater MAC protocol, new challenges that arise because of the unique characteristics of the underwater acoustic channel need to be carefully considered. In particular, the speed of sound under water is nearly 1500 m/s, which is five orders of magnitude lower than a radio signal's propagation speed of 3 × 10^8^ m/s. The underwater acoustic channel is also characterized by a narrow and low bandwidth that results in low data rates. Consequently, most terrestrial MAC protocols for wireless sensor networks (WSNs) cannot be directly applied in the underwater environment, because they are designed for supporting high data rates with negligible propagation delay.

Nonetheless, there have been some studies that have tried to apply the existing terrestrial MAC protocols to underwater environments. Under conditions of light traffic load, a purely uncontrolled random access protocol, such as Aloha, has a lower packet delay, because it transmits directly whenever a packet is generated. However, because of the lack of a collision avoidance mechanism, Aloha generates a significant number of collisions as the traffic load increases. The throughput analysis of Aloha in the underwater environment was presented in [3] and [4]. To reduce the collisions of Aloha, Nitthita *et al.* proposed two Aloha-based protocols, namely, Aloha with collision avoidance (Aloha-CA) and Aloha with advance notification (Aloha-AN) [5]. These two protocols utilize the information obtained from the overheard packets to calculate the busy durations of neighboring nodes and avoid collisions accordingly. Unlike Aloha-based protocols, the carrier sense multiple access (CSMA) [6] makes a node listen to the channel before transmitting a packet, that is, a node may start transmitting if and only if it senses that the channel is idle. However, in a long propagation delay environment, the carrier sensing cannot indicate the real status of the channel, which means that the carrier sensing mechanism is not appropriate for the underwater environment.

Current research efforts on underwater MAC protocols strongly focus on the handshaking-based MAC protocols that reserve a channel by exchanging control packets, such as request-to-send (RTS) and clear-to-send (CTS). Existing handshaking-based underwater MAC protocols can be categorized into two types: sender-initiated and receiver-initiated. The multiple-access collision avoidance (MACA) [7] is a popular and representative sender-initiated MAC protocol that uses the three-way RTS/CTS/DATA handshake. In MACA, an exchange of RTS and CTS between sender and receiver takes place prior to data transmission. Hence, neighbors overhearing the control packets can defer their communication in order to avoid possible collisions that are addressed as a hidden-node problem. However, in the underwater environment, the simple exchange of RTS and CTS barely solves the hidden-node problem because of the long propagation delay of the acoustic channel.

To overcome this problem, Molins and Stojanovic proposed slotted floor acquisition multiple access (Slotted-FAMA) [8] that combines both carrier sensing and RTS/CTS handshake mechanisms. In this protocol, packets are transmitted at the beginning of a slot whose length is equal to the maximum propagation delay. Although the Slotted-FAMA can prevent collisions caused by hidden nodes, the excessive slot length decreases the throughput performance. Like the Slotted-FAMA, the distance-aware collision avoidance protocol (DACAP) proposed in [9] combines carrier sensing and RTS/CTS handshake mechanisms, but the nodes need not be synchronized. This enables a sender to use different handshake lengths for different receivers to minimize the average handshake duration. In addition, DACAP waits some time before transmitting the data packet to guarantee the absence of harmful collisions.

Another CSMA-based MAC protocol, named propagation delay aware protocol (PDAP), was proposed in [10]. PDAP aims at maximizing the bandwidth utilization by keeping track of neighboring transmissions to avoid collisions, thus enabling interleaved packet transmission between different pairs of users. In order to solve the problem of space-time uncertainty, a new class of MAC protocol, called Tone Lohi (T-Lohi), was proposed in [11]. T-Lohi uses short contention tones to reserve the channel for competing nodes. This tone-based reservation mechanism provides collision avoidance and low energy consumption. However, T-Lohi requires a node to be idle and listen to the channel for every contention round when competing for the channel, and because the listening period lasts for at least the maximum propagation delay time plus the time to detect the contention tone, it results in a low channel utilization [12].

Among MACA-based protocols, MACA for underwater (MACA-U) [13] is the basic and reference protocol that revises the state transition rules that account for the long propagation delay. In [14], Liao and Huang proposed the spatially fair MAC (SF-MAC) protocol that concerns not only the collisions, but also the unfairness problem caused by the long propagation delay. SF-MAC prevents collisions by postponing the transmission of the CTS packet. The receiver collects RTS packets from all the potential senders during the RTS contention period and determines the earliest transmitter, achieving a higher degree of fairness. However, SF-MAC has a long, fixed RTS contention period, which critically affects channel utilization. To improve the channel utilization, Guo *et al.* proposed the adaptive propagation-delay-tolerant collision-avoidance protocol (APCAP) [15] that enables a sender to perform other functions during the large time gap between the transmission of RTS and the corresponding CTS reception, which is called MAC level pipelining. However, APCAP requires a time synchronization and a complicated process for MAC level pipelining.

A delay-aware opportunistic transmission scheduling (DOTS) protocol [16] uses passively obtained local information (neighboring nodes' propagation delay map) to increase the chances of concurrent transmissions while reducing the likelihood of collisions. Another way to improve the channel utilization is a packet-train approach. Chirdchoo *et al.* proposed a MACA-based MAC protocol with packet-train to multiple neighbors (MACA-MN) [17]. MACA-MN improves channel utilization by sending multiple packets to multiple neighbors in each round of handshake. MACA-U with packet trains (MACA-UPT) was also introduced in [18]. MACA-UPT is derived from MACA-U, except that a sender transmits multiple data packets in a single handshake in the former. Recently, Hai-Heng Ng *et al.* proposed a bidirectional concurrent MAC (BiC-MAC) protocol [18], wherein a sender-receiver pair simultaneously transmits data packets to each other, which improves the channel utilization. Hai-Heng Ng *et al.* also proposed a MAC protocol using reverse opportunistic packet appending (ROPA) [19], which is a hybrid of sender-initiated and receiver-initiated MAC protocols. ROPA improves channel utilization by enabling a sender to coordinate multiple neighbors to opportunistically transmit (append) their data packets. After the sender finishes transmitting its data packets, it starts to receive incoming appended data packets. However, in ROPA, more control packet exchange is needed; therefore, more collisions may occur.

On the other hand, in [20], Chirdchoo *et al.* proposed the receiver-initiated packet train (RIPT) protocol that falls into the category of the receiver-initiated MAC protocols. When a node wishes to become a receiver, it initiates the four-way ready-to-receive (RTR)/SIZE/ORDER/DATA handshake that schedules the packets from multiple neighbors to arrive at the receiver in a packet train. Although RIPT can get multiple data packets from neighbors, the four-way handshake takes a long time to receive the first packet train at the receiver node, especially in the underwater environment.

As described above, the long propagation delay, which is a major feature to be considered in the case of underwater acoustic channels, makes it difficult to design underwater MAC protocols. In particular, in handshaking-based MAC protocols, the exchange of control packets is time-consuming, resulting in a large signaling overhead. Furthermore, in the case of multi-hop relaying in a hop-by-hop handshaking manner, the end-to-end delay is significantly increased. Therefore, this paper proposes a new underwater MAC protocol, named cascading multi-hop reservation and transmission (CMRT), to address the abovementioned problems. The CMRT protocol reserves the multi-hop channels at once by cascading reservation control packets and delivers the data packets in the same way until they reach the destination without stopping at intermediate nodes. This multi-hop reservation approach is different from what conventional MAC protocols employ for multi-hop transmission as explained above. In addition, CMRT adopts a packet-train method [17] to improve channel utilization by sending multiple data packets together with only one handshaking signal. In this way, CMRT is able to reduce the control packet exchange time and accordingly increase the throughput compared with conventional MAC protocols. The main contributions of this paper can be summarized as follows:

1. Propose a cascading multi-hop reservation-based MAC protocol for UWSNs with a long propagation delay to significantly reduce the end-to-end delay and improve channel utilization.
2. Compare the performance with conventional MAC protocols in terms of throughput and end-to-end delay.
3. Propose a new RTS attempt triggering method that adaptively changes the batch size of data packets transmitted with a single reservation.

The rest of the paper is organized as follows. Section 2 presents the problem statements. In Section 3, we explain the proposed protocol design, including a new RTS attempt strategy. We present simulation results and their discussions in detail in Section 4. Finally, the conclusions are provided in Section 5.

## 2. Problem Statements

The long propagation delay of the underwater acoustic channel poses challenges for the design of MAC protocols, such as space-time uncertainty and the hidden-node problem. Furthermore, the end-to-end delay is substantially increased in multi-hop relaying. In this section, we describe these problems in detail.

### 2.1. Space-Time Uncertainty

**Figure 1.**
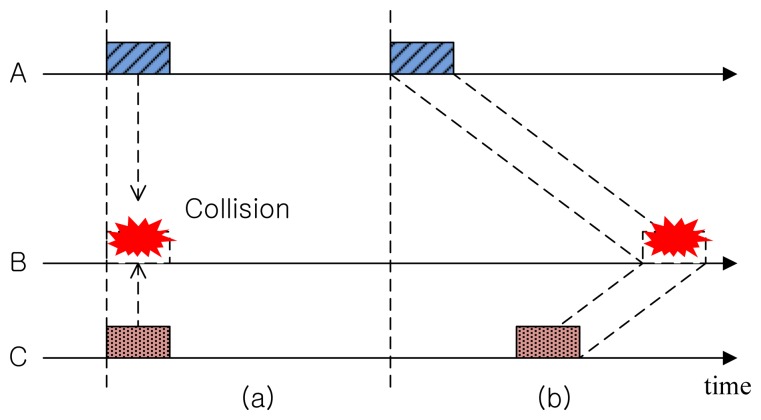
Space-time uncertainty: (**a**) terrestrial RF channel; and (**b**) underwater acoustic channel.

Figure 1a illustrates a collision that occurs in RF-based terrestrial WSNs where the propagation delay is negligible and the y-axis denotes the distance between nodes. When Nodes A and C are transmitting packets at the same time, the packets collide at destination Node B. Such collisions can be avoided by scheduling in such a way that the durations of the transmission time do not overlap. That is, we have to consider only the transmission time uncertainty.

On the other hand, in the case of UWSNs, the long propagation delay of the acoustic signal makes it more complicated to avoid any collisions, because we have to consider not only the transmission time, but also the distance (space) between nodes. Figure 1b shows an example where two packets transmitted from Nodes A and C at different times collide at Node B. We call such a two-dimensional uncertainty in determining a collision at the receiver as space-time uncertainty [4].

### 2.2. Hidden-Node Problem in UWSNs

**Figure 2.**
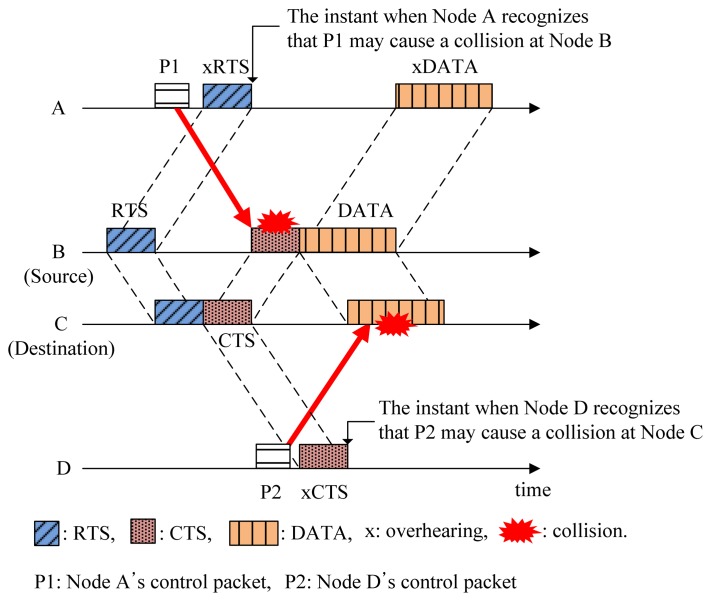
Hidden-node problem in the underwater acoustic channel. RTS, request-to-send; CTS, clear-to-send.

In conventional handshaking protocols for collision avoidance (CA), the source node makes a channel reservation by sending an RTS control packet. The destination node replies to the RTS with a CTS control packet that can be overheard by neighbors (potential interferers), so that they recognize that the channel will be reserved during a certain amount of time. Accordingly, the source node can transmit data packets to the destination node without collisions. This is the basic method adopted in CA protocols for preventing possible collisions caused by hidden nodes. However, the long propagation delay in the underwater acoustic channel introduces a new kind of hidden-node problem, as shown in Figure 2.

In the underwater acoustic channel, some nodes may detect the channel reservation after transmitting control packets (e.g., P1 and P2 in Figure 2). This may cause possible collisions at source and destination nodes as denoted by the solid arrows in Figure 2. We call this unexpected collision caused by the long propagation delay the hidden-node problem in the underwater acoustic channel. In Figure 2, Nodes A and D become hidden nodes.

## 3. Proposed CMRT Protocol

### 3.1. System Description

We consider a multi-hop network where all nodes are equipped with half-duplex and omni-directional acoustic modems. It is assumed that every node knows the inter-nodal distance to its neighbors within a one-hop range and keeps a list of those with which it can establish a bi-directional link. During the network initialization phase, the inter-nodal distance is obtained by using round-trip time (RTT) measurements of control packets or by sharing some information among neighbors [21]. It is also assumed that every node has the routing table to facilitate multi-hop relay.

#### 3.1.1. Definition of States

In CMRT, a node shifts between six different states, namely, Idle, Wait_Resp (Wait for RESPonse), Delay_Data (Delay Data transmission), Wait_Data (Wait for Data reception), Data_Rx (Data Reception) and Silence.

**Figure 3.**
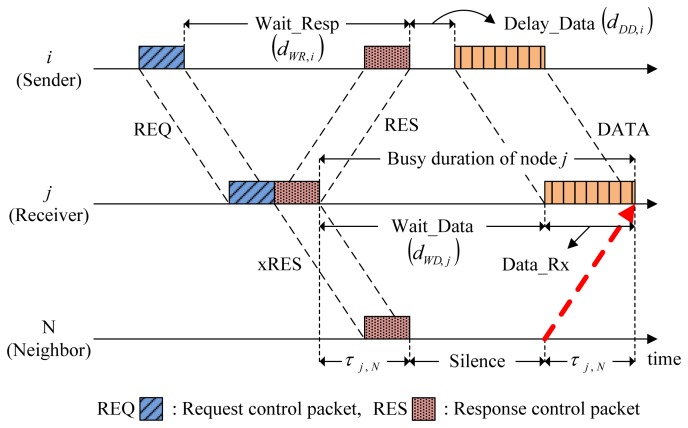
The six different states of a node.

Figure 3 illustrates the individual states that may occur in the CMRT procedure.

1. Wait_Resp is a state where a sender waits for a response to a request control packet (e.g., RTS) from a receiver. The sender stays in the Wait_Resp state directly after transmitting a request control packet until receiving a response control packet (e.g., CTS). If the sender does not receive a response control packet within the duration of Wait_Resp state, it will transit to the Idle state.
2. Delay_Data is a state where a sender delays data transmission to avoid possible collisions caused by the hidden nodes. After receiving a response control packet from the receiver, the sender enters the Delay_Data state and remains there until it starts transmitting data packets. The length of the Delay_Data state should be elaborately calculated, and the calculation procedure will be presented in Section 3.2.
3. Wait_Data is a state where a receiver waits for data packets from a sender. The receiver enters the Wait_Data state directly after transmitting a response control packet and remains there until it starts receiving data-packets.
4. Data_Rx is a state where a receiver receives data packets.
5. Silence is a state where neighbors who overheard the exchange of control packets for channel reservation remain silent, doing nothing so that they do not cause collisions. Neighbors enter the Silence state after overhearing the control packets involved in other nodes' channel reservation until the channel becomes free of reservation. The Silence state ensures that any transmissions from neighbors arrive after data reception is completed at a receiver, as denoted by the dotted arrow in Figure 3.
6. The Idle state includes the remaining cases not belonging to the five states described above.

The length of each state that indicates the time duration of node i staying in the corresponding state is listed in Table 1.

#### 3.1.2. Length of the Silence State

First, we define the busy duration as an interval between when a control packet is sent to neighbors and when a responding control packet (e.g., CTS) or a data packet is received as a reply from the neighbors. In the case of a sender (node *i* in Figure 3), the reply is carried out by a responding control packet. Thus, the busy duration of node *i* is given by the interval between the end of REQ (request) transmission and the end of RES (respond) reception as:

(1) $$d_{\text{busy},i}=d_{WR,i}$$

On the other hand, in the case of a receiver or a relay (node *j* in Figure 3), the reply is carried by a data packet. Thus, as shown in the Figure 3, the busy duration of node *j* includes not only the Wait_Data, but also the data reception time denoted by *d_DATA_* as:

(2) $$d_{\text{busy},j}=d_{WD,j}+d_{\text{DATA}}$$

Every node specifies its busy duration inside the control packets. For example, in Figure 3, REQ and RES contain the busy durations for node *i* and *j*, respectively. Overhearing RES from node *j*, its neighbor, Node N, can easily calculate the length of Silence from:

(3) $$d_{\text{silence}}=d_{\text{busy},j}-2\tau_{j,N}$$

where *τ_i,j_* is the propagation delay between nodes *i* and *j*. In the same way, all neighbors receive information regarding how long they have to stay in Silence. Whenever a node in Silence overhears another neighbor's control packet, it extends its Silence duration accordingly.

#### 3.1.3. Channel Occupancy Priority

In general, relay nodes handle two types of data packets: those generated by themselves, called domestic data packets, and those relayed from the neighbors, called foreign data packets. It is assumed that a foreign data packet has priority to occupy the channel over a domestic data packet. Such a policy is named the foreign-first policy. Each node manages two separate buffers, one each for domestic and foreign data packets. Let *N^i^*^→^*^j^* be the number of data packets destined for node *j* and stored in the buffer of node *i*. Now,

(4) $$N^{i\to j}=N_{\text{dom}}^{i\to j}+N_{\text{frg}}^{i\to j}$$

where $N_{\text{dom}}^{i\to j}$ and $N_{\text{frg}}^{i\to j}$ are the numbers of domestic and foreign data packets destined for node *j* and stored in the buffer of node *i*, respectively. *N^i^*^→^*^j^* has a limited capacity of *N*_max_. In terms of priority among foreign data packets, those belonging to the dominant set that contains a group of data packets destined to node *k*, such that $\underset{k}{\arg}\max\left[N_{\text{frg}}^{i\to k}\right]$, are transmitted first. This priority policy is named the dominant-first. Furthermore, inside the dominant set, data packets that have traveled by the largest number of hops until that instant are given the highest priority to occupy the channel. This priority policy is named the oldest-first.

### 3.2. Cascading Multi-Hop Reservation

**Figure 4.**
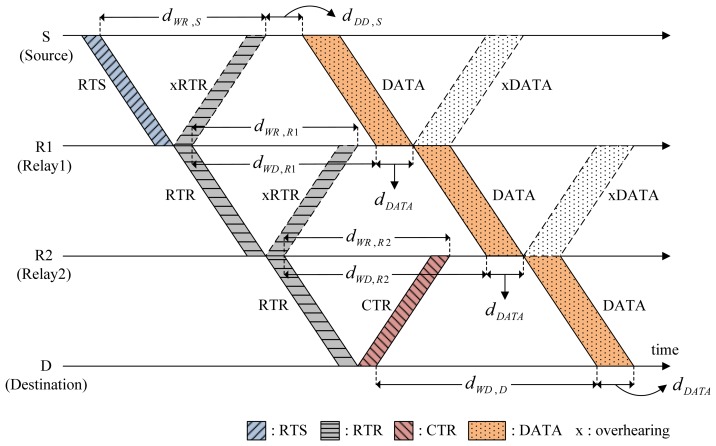
Operation of the cascading multi-hop reservation and transmission (CMRT) protocol.

Figure 4 shows a scenario of CMRT operation. It is assumed that two relays R1 and R2 exist between source S and destination D. A multi-hop relay begins with the source S staying in the Idle state by transmitting RTS to relay R1. After transmitting RTS, Node S enters the Wait_Resp state. The RTS packet contains the following information: (1) the address of the final destination (FD), Node D in this example; (2) batch size, the number of data packets to be transmitted, *B_size_*; (3) the busy duration of Node S, *d_busy,S_*; and (4) hop count to denote the number of hops from the source node, *k_S_*. The value of the hop count will be increased by one as the channel reservation progresses.

Upon receiving RTS, the relay node R1 transmits a control packet named request-to-reserve (RTR) to the next node in order to reserve the channel for the next hop. Here, RTR is a newly introduced control packet in CMRT and is paired with a responding control packet named clear-to-reserve (CTR) similar to the pairing of RTS with CTS. The RTR packet also contains the same information as RTS, [*FD, B_size_, d_busy,R_*_1_, *k_R_*_1_], where *k_R1_* = *k_S_* + 1. The RTR is used not only to reserve the channel for the next hop, but also to respond to RTS/RTR of the previous hop to allow backward overhearing. In Figure 4, when Node R1 relays RTR to Node R2 in the forward direction, Node S overhears RTR in the backward direction, which is denoted by xRTR, to recognize that the node's previous request (RTS) was successfully sent and processed for the next-hop relay. After relaying RTR, Node R1 enters Wait_Resp and Wait_Data states at the same time. Accordingly, unlike a sender (Node S), Node R1 would not transit to the Idle state immediately, even if it does not receive a response control packet (xRTR) from node R2 within the duration of Wait_Resp state. Instead, Node R1 will stop the data forwarding and transit to the Idle state after the Data_Rx state regardless of whether it successfully receives a train of data packets. All relay nodes work in the same manner as Node R1. Destination D stops relaying RTR and instead transmits CTR to the previous relay node as a response to RTR. The CTR packet (as well as the CTS packet) contains the information about the busy duration of destination D (*d_busy,D_*). Note that the RTR plays a key role here for cascading reservation information through multiple hops, thus efficiently reducing handshaking and data delivery times.

**Figure 5.**
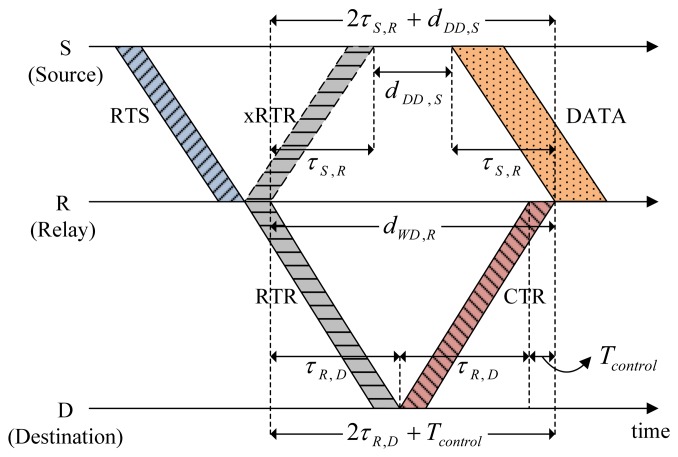
Determination of *d_DD,S_*.

Source S delays data transmission for the length of Delay_Data (*d_DD,S_*) to avoid causing possible collisions with the hidden nodes. For the simple case of two-hop relaying (source → relay, relay → destination) illustrated in Figure 5, the following relation between timing parameters is obtained:

(5) $$2\tau_{S,R}+d_{DD,S}=2\tau_{R,D}+T_{\text{control}}$$

where *T_control_* is the common transmission time of all control packets. Let *τ*_max_ be the maximum propagation delay between nodes that corresponds to the transmission range. The length of Delay_Data for the worst case (*τ_R,D_* = *τ*_max_) is now obtained as:

(6) $$d_{DD,S}=2\left(\tau_{\max}-\tau_{S,R}\right)+T_{\text{control}}$$

### 3.3. Data Transmission Using the Packet-Train Method

**Figure 6.**
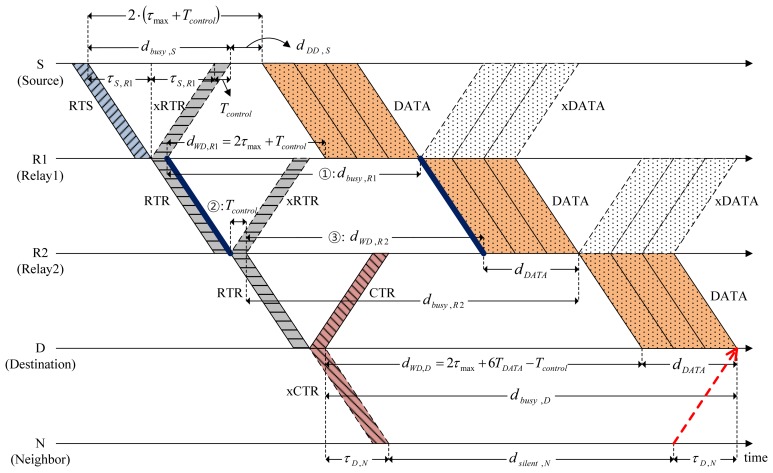
Data transmission using a packet train.

To increase channel utilization, CMRT adopts a packet-train method [17] where multiple data packets are sent in a row by handshaking once. Figure 6 illustrates how the packet-train method is used in CMRT for the case of *B_size_* = 3. Source S sequentially transmits a train of data packets to the next relay node without any interval between packets. Similarly, relay Nodes R1 and R2 also forward the train without delay because the multi-hop channels to the destination are already reserved. If a relay node does not receive a train of data packets within the duration of the Data_Rx state, it would transit to the Idle state after the Data_Rx state ends.

Depending on the batch size, the busy duration for the different types of nodes, namely, source, relay and destination, should be determined. On the basis of Equation (1), the busy duration of Node S in Figure 6 is given by:

(7) $$d_{\text{busy},S}=2\tau_{S,R1}+T_{\text{control}}$$

On the basis of Equation (2), the busy duration of R1 is:

(8) $$d_{\text{busy},R1}=d_{WD,R1}+d_{\text{DATA}}$$

where *d_WD,R1_* is obtained from Figure 5 and Equation (6) by:

(9) $$\begin{array}{ll} d_{WD,R1} & =2\tau_{S,R1}+d_{DD,S}\\ & =2\tau_{S,R1}+2\left(\tau_{\max}-\tau_{S,R1}\right)+T_{\text{control}}\\ & =2\tau_{\max}+T_{\text{control}} \end{array}$$

and:

(10) $$d_{\text{DATA}}=B_{\text{size}}\cdot T_{\text{DATA}}$$

where *T_DATA_* is the transmission time of a single data packet. In the same way as Node R1, the busy duration of R2 is:

(11) $$d_{\text{busy},R2}=d_{WD,R2}+d_{\text{DATA}}$$

where the length of Wait_Data of R2, *d_WD,R2_* (denoted by ➂ in Figure 6), is directly obtained from *d_busy,R_*_1_ (denoted by ➀, the time duration between the two bold lines in Figure 6) and *T_control_* (denoted by ➁). That is,

(12) $$d_{WD,R2}=d_{\text{busy},R1}-T_{\text{control}}(➂=➀-➁)$$

Substituting Equations (10) and (12) for Equation (11), the busy duration of R2 is finally given by:

(13) $$\begin{array}{ll} d_{\text{busy},R2} & =\left(d_{\text{busy},R1}-T_{\text{control}}\right)+B_{\text{size}}\cdot T_{\text{DATA}}\\ & =2\tau_{\max}+2B_{\text{size}}\cdot T_{\text{DATA}} \end{array}$$

In the same way, we can generalize the busy duration of relay node *Ri* as:

(14) $$\begin{array}{ll} d_{\text{busy},Ri} & =d_{WD,Ri}+d_{\text{DATA}}\\ & =\left(d_{\text{busy},Ri-1}-T_{\text{control}}\right)+B_{\text{size}}\cdot T_{\text{DATA}}\\ & =2\tau_{\max}+i\cdot B_{\text{size}}\cdot T_{\text{DATA}}-(i-2)\cdot T_{\text{control}} \end{array}$$

where the value of *i* can be determined from the hop-count included in RTS/RTR packets. Similarly, the busy duration of the destination when *k* relay nodes exist between the source and destination nodes is given by:

(15) $$d_{\text{busy},D}=2\tau_{\max}+\left(k+1\right)\cdot B_{\text{size}}\cdot T_{\text{DATA}}-(k-1)\cdot T_{\text{control}}$$

### 3.4. Handshaking Triggering and Back-Off Algorithm

In conventional CA protocols, each node starts the handshaking procedure under two types of conditions: (1) when the buffer becomes full (batch-by-size); and (2) when a predefined timer is expired (batch-by-time). In [18], a hybrid scheme combining “batch-by-size” and “batch-by-time” conditions was used. In the batch-by-size scheme, the proper value of the batch size (*B_size_*) depends on traffic conditions. If *B_size_* is too large under light traffic, the node spends too much time in waiting until the buffer becomes full. On the other hand, if *B_size_* is too small under heavy traffic, the node tries handshaking too frequently, resulting in a heavy signaling load. In CMRT, we propose a new scheme named “batch-by-adaptive-size” where *B_size_* of a given node *I* is adaptively changed according to the traffic condition as:

(16) $$B_{\text{size}}=N^{i\to j}$$

where:

(17) $$j=\underset{k}{\arg\max}\left(N^{i\to k}\right)$$

A batch-by-adaptive-size scheme is capable of working adaptively under varying traffic load conditions.

For RTS trials, CMRT adopts the binary exponential back-off (BEB) algorithm specified in the IEEE 802.11 standard [22]. In the BEB algorithm, a sender doubles its back-off counter (*B_cnt_*) with the upper bounds of *B_max_* when an RTS fails. On the other hand, upon successful transmission, a node resets its back-off counter to the minimum value of *B_min_*. The duration of the back-off is selected randomly in the range of zero to the back-off interval (*B_interval_*), which can be expressed as:

(18) $$B_{\mathrm{int}\text{erval}}=\text{random}\left(0,B_{\text{cnt}}\right)\times \tau_{\max}$$

## 4. Simulations and Results

### 4.1. Simulation Model

**Figure 7.**
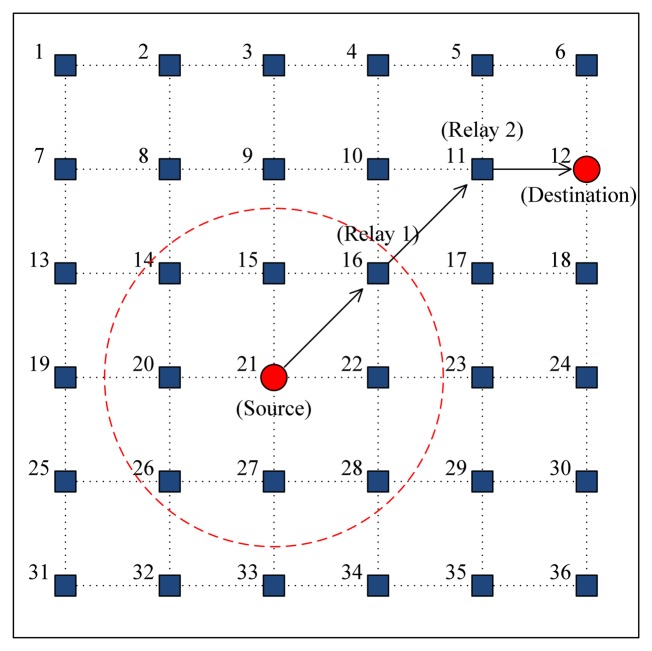
The network topology for simulations.

An event-driven network simulator was developed using MATLAB. As shown in Figure 7, a multi-hop topology is considered to have 36 static nodes placed in a 5000 × 5000 m^2^ square area with a grid spacing of 1000 m. All of the nodes are assumed to have the same transmission power and, accordingly, the same transmission range (1.5-times the grid spacing), such that each node has exactly eight neighbors within its range (the dotted circle in Figure 7). Each node generates data packets according to the Poisson process with an arrival rate λ_node_ (packets/s) and randomly selects a destination with equal probability. For multi-hop transmission, we apply the static routing where each node uses a manually configured routing entry. The acoustic channel is assumed to be error-free; that is, packet losses occur only in the case of packet collisions. The system parameters for simulation are summarized in Table 2. The transmission rate is referenced from the LinkQuest medium range acoustic mode [23].

### 4.2. Simulation Results

**Figure 8.**
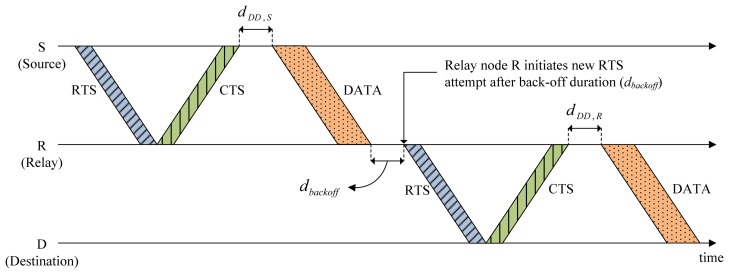
Operation of CMRT-S (single hop).

The CMRT is compared with the two conventional MAC protocols, MACA-U [13] and MACA-UPT [18], and the single-hop repeated version of CMRT (CMRT-S) in terms of the normalized throughput per node and end-to-end packet delay. CMRT-S is a modified version of CMRT, where a single-hop transmission of CMRT is repeated multiple times in a hop-by-hop manner until the destination, as shown in Figure 8. Unlike the hop-by-hop application of previous protocols, such as MACA-U and MACA-UPT, CMRT-S uses the collision-escape mechanism that originated from CMRT, according to which after receiving a CTS, the source waits for a certain amount of time before transmitting data packets.

The normalized throughput per node is defined as:

(19) $$\gamma =\frac{1}{N}\cdot \frac{\overset{N}{\underset{i=1}{\text{∑}}}r_{i}\cdot B_{\text{DATA}}}{t_{\text{sim}}}$$

where *B_Data_* is the size of a data packet in bits and *N* is the total number of nodes in the network. Depending on the position along the data-packet relay route, the node could be a source, a relay or a destination. *r_i_* is the number of data packets successfully received by destination node *i*, and *t_sim_* is the simulation time. As another performance measure, the end-to-end packet delay is defined as the time duration from when a data packet is generated at a source to when it is successfully received at a destination. Let Ω be the set of data packets that arrive successfully at the destination. The size of Ω is given by $N\left(\Omega \right)=\overset{N}{\underset{i=1}{\text{∑}}}r_{i}$, and each element of Ω has a different end-to-end packet delay, *t_delay,j_, j* = 1,2, … ,*N*(Ω). Thus, the average end-to-end packet delay is defined as:

(20) $$\bar{t_{\text{delay}}}=\frac{\overset{N\left(\Omega \right)}{\underset{j=1}{\text{∑}}}t_{\text{delay},j}}{N\left(\Omega \right)}$$

Regarding the channel occupancy priority, the three policies of foreign-first, dominant-first, and oldest-first described in Section 3.1.3 are applied to all protocols for comparison.

#### 4.2.1. Comparison of CMRT with Other MAC Protocols

**Figure 9.**
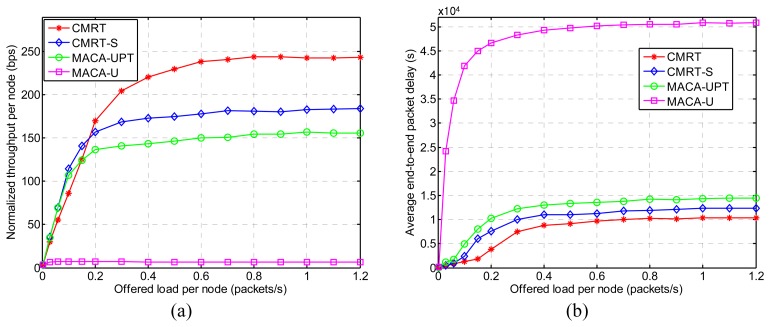
Performance comparisons of CMRT with other MAC protocols: (**a**) normalized throughput per node, and (**b**) average end-to-end packet delay. MACA-UPT, multiple-access collision avoidance for underwater with packet trains.

Figure 9a shows the normalized throughput per node (hereafter referred to as throughput) for various offered loads per node (λ_node_, hereafter referred to as offered load), and Figure 9b shows the average end-to-end packet delay (hereafter referred to as delay) performance. In most cases of the offered load, CMRT exhibits the best performance in terms of both throughput and delay. That is because CMRT is able to significantly reduce the time spent in handshaking and data transmission by means of cascading channel-reservation and data-transmission over multiple hops and the packet-train method. Additionally, CMRT considers the hidden-node problem when scheduling the transmission time. Unlike CMRT, the three other protocols (CMRT-S, MACA-UPT and MACA-U) conduct multi-hop transmission by adopting the conventional way of hop-by-hop relaying, in which the next-hop relay starts only after completion of the previous-hop relay. The reason CMRT-S exhibits better performance than MACA-UPT and MACA-U is that CMRT-S is capable of handling the hidden-node problem by postponing the data transmission after receiving a CTS. MACA-U, which does not use the packet-train method, exhibits the worst performance. The features of the schemes aforementioned are summarized in Table 3.

On the other hand, when the offered load is lower than 0.2, the throughput of CMRT is slightly lower than that of CMRT-S and MACA-UPT. In CMRT, when a node is involved in multi-hop relaying as a relay node, the relay node is allowed to send only foreign data-packets and not domestic data-packets, even if it has domestic ones, in order to maintain the constant size of the data stream from source to destination. On the other hand, in hop-by-hop relaying schemes, such as CMRT-S and MACA-UPT, every hop is refreshed, so that a sender plays the role of a source all of the time and sends as many foreign and domestic data packets as possible. Thus, under a light traffic condition, CMRT does not have enough data packets to fully achieve its capability. Saturation of both the throughput and the delay with the increase in the offered load is caused by the limited buffer capacity of 300 data packets (refer to Table 2).

**Figure 10.**
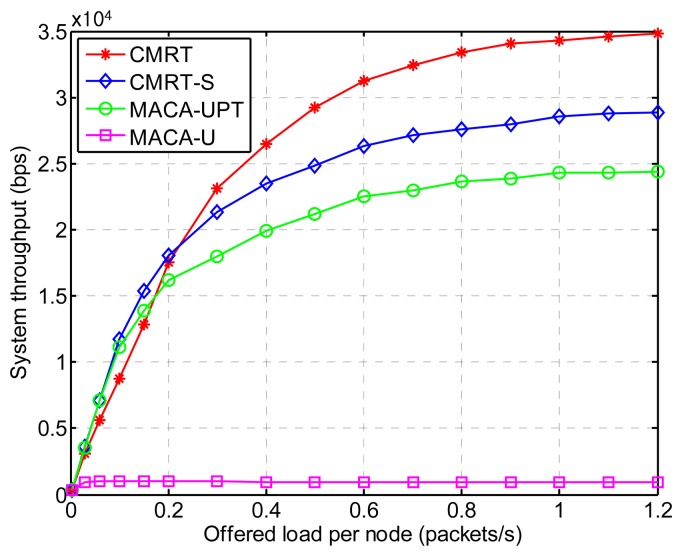
System throughput of CMRT in comparison with other MAC protocols.

Figure 10 shows the system throughput *versus* the offered load, which is defined as follows:

(21) $$S=\frac{\overset{N}{\underset{i=1}{\text{∑}}}s_{i}\cdot B_{\text{Data}}}{t_{\text{sim}}}$$

where *s_i_* is the total number of data packets successfully received by node *i*. Therefore, the system throughput includes successfully received data packets by not only final destinations, but also relay nodes. Consequently, the system throughput means the overall channel utilization by using the MAC protocol. Thus, similar to the case of normalized throughput per node, CMRT outperforms other alternatives in terms of the channel utilization.

#### 4.2.2. Analysis of Hop-Delay

**Figure 11.**
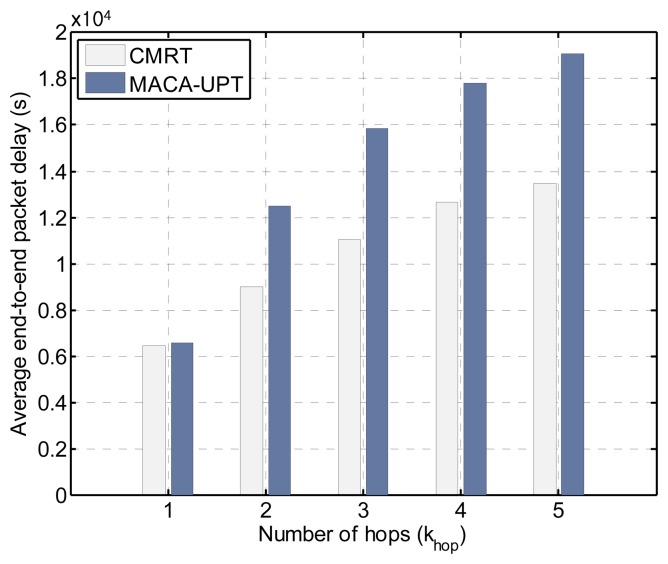
Delay comparison between CMRT and MACA-UPT by varying the number of hops between source and destination, *k_hop_*.

To provide further insight into the performance of CMRT, we analyze the delay (shown in Figure 9b) in more detail according to the number of hops between source and destination nodes that is denoted by *k_hop_*. Figure 11 shows the delay of CMRT and MACA-UPT *versus k_hop_*, when the offered load is fixed at 1.0 packets/s, which is large enough to ensure that a node always has data packets in its buffer. In our simulation model, where 36 static nodes are located in the grid of a square area, as shown in Figure 7, *k_hop_* varies from one to a maximum of five. As *k_hop_* increases, the difference between the delays with CMRT and MACA-UPT becomes larger, because the gain of cascading transmission is cumulative. Note that the incremental delay is not proportional to *k_hop_* owing to the priority policy of oldest-first. That is, at the instant of a priority decision, the data packets with a larger *k_hop_* are likely to be selected as the oldest packets that have traveled through the largest number of hops.

#### 4.2.3. Effects of Inter-Nodal Distance

**Figure 12.**
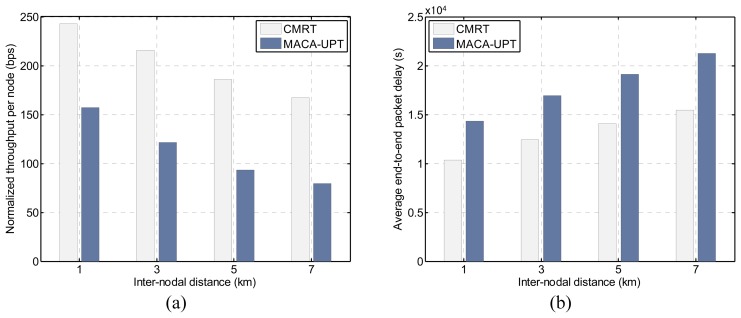
Effects of inter-nodal distance on CMRT and MACA-UPT: (**a**) normalized throughput per node, and (**b**) average end-to-end packet delay.

Figure 12a,b shows the effects of inter-nodal distances on the throughput and the delay, under the offered load of 1.0 packets/s. As the inter-nodal distance increases, the performance of each of the protocols in terms of both throughput and delay deteriorates because the distance-related communication overhead increases accordingly. The increase in the propagation delay due to the extended distance causes an increase in the busy duration, as well as the handshaking time. Consequently, the prolonged busy duration increases the length of the Silence state, and thus, a node has less opportunity to attempt an RTS. However, we have shown that compared with MACA-UPT, CMRT could be a better solution for use in more scalable multi-hop networks.

## 5. Conclusions

This paper has discussed the challenges posed by the long propagation delay in the underwater acoustic channel that need to be considered when designing an underwater channel MAC protocol and achieving the benefits of multi-hop relay. On the basis of these considerations, a cascading multi-hop reservation and transmission MAC protocol named CMRT has been proposed. To reduce the time-related overhead caused by the propagation delay, CMRT makes a relay node start handshaking for the next hop, while the handshaking for the previous hop is in progress. With this concurrent relaying, the flow of control and data packets starting from a source cascades to the destination without stopping at any relay nodes. In addition, CMRT is able to reduce the control packet exchange time by utilizing the backward overhearing of a control packet forwarded by the next hop relay node as a response. To prevent unexpected collisions caused by the hidden-node problem, CMRT postpones data transmission until potential interferers enter the silent mode by recognizing the channel to be reserved. Furthermore, to improve channel utilization, CMRT adopts a packet-train method. Computer simulation shows that CMRT outperforms other well-known underwater MAC protocols, such as MACA-U and MUAC-UPT in terms of both throughput and delay. In further works, the following problems will be investigated: (1) a time-efficient acknowledgment scheme; (2) improvement of spatial fairness between sensor nodes; and (3) protocol evaluation under more realistic underwater acoustic channel conditions.

## Figures and Tables

**Table 1.** Description of the length of each state.

**Notation**	**Description**
*d_WR,i_*	Length of the Wait_Resp for node *i*
*d_DD,i_*	Length of the Delay_Data for node *i*
*d_WD,i_*	Length of the Wait_Data for node *i*

**Table 2.** System parameters.

**Parameter**	**Value**
Transmission rate	9600 bps
Size of data packet	1200 bits
Size of control packet	120 bits
Minimum back-off counter (*B_min_*)	1
Maximum back-off counter (*B_max_*)	64
Capacity of buffer (*N_max_*)	300 packets
Acoustic propagation speed	1500 m/s

**Table 3.** Comparison of the features of MAC protocols.

**Feature**	**Cascading Reservation and Transmission**	**Packet-Train Method**	**Solution for Hidden-Node Problem**

**MAC Protocol**			
CMRT	O	O	O
CMRT-S	X	O	O
MACA-UPT	X	O	X
MACA-U	X	X	X
